# Supplementary material for: AT Homopolymer Strings in Salmonella enterica Subspecies I Contribute to Speciation and Serovar Diversity
Source: Microorganisms. 2021 Oct 1;9(10):2075. doi: 10.3390/microorganisms9102075 (PMC8538453; doi:10.3390/microorganisms9102075)
Supplement: Supplementary file 1 [file microorganisms-09-02075-s001.zip › Table S1 V1 - List of strains.pdf]

TABLE S1. List of bacterial genomes analyzed for kmer homopolymers

| *Salmonella enterica* subspecies I serovar strain | NCBI reference | Other genera | NCBI reference |
|---|---|---|---|
| Salmonella enterica subspecies I serovar Agona | NC_011149.1 | Bacillus anthracis Ames ancestor | NC_007530.2 |
| Salmonella enterica subspecies I serovar Braenderup | CP022490.1 | Bacillus anthracis CDC684 | NC_012581.1 |
| Salmonella enterica subspecies I serovar Enteritidis ATCC BAA-708 | CP025554.1 | Bacillus anthracis Sterne | NC_005945.1 |
| Salmonella enterica subspecies I serovar Enteritidis CDC_2010K_0968 | CP007528.1 | Bacillus cereus ATCC14579 | NC_004722.1 |
| Salmonella enterica subspecies I serovar Enteritidis CFSAN018747 | CP028196.1 | Bacillus cereus B4264 | NC_011725.1 |
| Salmonella enterica subspecies I serovar Enteritidis CFSAN033543 | CP020825.1 | Bacillus cereus G9842 | NC_011772.1 |
| Salmonella enterica subspecies I serovar Enteritidis CFSAN051873 | CP022003.1 | Enterococcus faecalis D32 | NC_018221.1 |
| Salmonella enterica subspecies I serovar Enteritidis CFSAN076214 | CP033340.1 | Enterococcus faecalis TY1 | CP031027.1 |
| Salmonella enterica subspecies I serovar Enteritidis EC20090641 | CP007249.2 | Enterococcus faecalis V583 | NC_004668.1 |
| Salmonella enterica subspecies I serovar Enteritidis FORC_052 | CP016754.1 | Escherichia coli CFSAN029787 | CP011416.1 |
| Salmonella enterica subspecies I serovar Enteritidis OLF-SE1-1019-1 | CP009083.1 | Escherichia coli Combat2C1 | CP019243.1 |
| Salmonella enterica subspecies I serovar Enteritidis P125109 | NC_011294.1 | Escherichia coli IAI39 | NC_011750.1 |
| Salmonella enterica subspecies I serovar Enteritidis strDurban | CP007507.1 | Escherichia coli JJ2434 | CP013835.1 |
| Salmonella enterica subspecies I serovar Enteritidis strRM2968 | CP028151.1 | Escherichia coli K-12 | NC_000913.3 |
| Salmonella enterica subspecies I serovar Gallinarum 9184 | CP019035.1 | Escherichia coli NCTC11151 | LR134031.1 |
| Salmonella enterica subspecies I serovar Heidelberg | NC_021812.2 | Escherichia coli Nissle 1917 | CP007799.1 |
| Salmonella enterica subspecies I serovar Infantis | CP019202.1 | Escherichia coli O104:H4 | NC_018658.1 |
| Salmonella enterica subspecies I serovar Javiana | NC_020307.1 | Escherichia coli O157:H7 | NC_002695.2 |
| Salmonella enterica subspecies I serovar Mbandaka | CP033343.1 | Escherichia coli O83:H1 | NC_017634.1 |
| Salmonella enterica subspecies I serovar Montevideo | CP029336.1 | Escherichia coli UK_Dog_Liverpool | CP031653.1 |
| Salmonella enterica subspecies I serovar Newport | NC_011080.1 | Escherichia coli UMN026 | NC_011751.1 |
| Salmonella enterica subspecies I serovar Oranienburg | CP019197.1 | Proteus mirabilis AR379 | CP029133.1 |
| Salmonella enterica subspecies I serovar Saintpaul | CP019206 | Proteus mirabilis HI4320 | NC_010554.1 |
| Salmonella enterica subspecies I serovar Schwarzengrund | NC_011094.1 | Proteus mirabilis VAC | CP042907.1 |
| Salmonella enterica subspecies I serovar Thompson | NC_022525.1 | Shigella sonnei 53G | NC_016822.1 |
| Salmonella enterica subspecies I serovar Typhi 129-0238-M | LT904888.1 | Shigella sonnei ATCC29930 | CP026802.1 |
| Salmonella enterica subspecies I serovar Typhi 311189_282186 | CP029920.1 | Shigella sonnei Ss046 | NC_007384.1 |
| Salmonella enterica subspecies I serovar Typhi 343077_214162 | CP029862.1 | Staphylococcus aureus Bmb9393 | NC_021670.1 |
| Salmonella enterica subspecies I serovar Typhi B/SF/13/03/195 | CP012151.1 | Staphylococcus aureus NCTC 8325 | NC_007795.1 |
| Salmonella enterica subspecies I serovar Typhi CT18 | NC_003198.1 | Staphylococcus aureus TW20 | NC_017331 |
| Salmonella enterica subspecies I serovar Typhi ERL024120 | LT905088.1 | Streptococcus pyogenes M1GAS | NC_002737.2 |
| Salmonella enterica subspecies I serovar Typhi P-stx-12 | NC_016832.1 | Streptococcus pyogenes MGAS10270 | NC_008024.1 |
| Salmonella enterica subspecies I serovar Typhi SGB92 | LT904877.1 | Streptococcus pyogenes MGAS6180 | NC_007296.2 |
| Salmonella enterica subspecies I serovar Typhi Ty21a | NC_021176.1 | Vibrio vulnificus CECT4999 cI | CP014636.1 |
| Salmonella enterica subspecies I serovar Typhi ty3-243 | LT905090.1 | Vibrio vulnificus CECT4999 cII | CP014637.1 |
| Salmonella enterica subspecies I serovar Typhi TY585 | LT904852.1 | Vibrio vulnificus Env1 cI | CP017635.1 |
| Salmonella enterica subspecies I serovar Typhi WGS1146 | CP040575.1 | Vibrio vulnificus Env1 cII | CP017636.1 |
| Salmonella enterica subspecies I serovar Typhimurium D23580 | NC_016854.1 | Vibrio vulnificus YJ016 cI | NC_005139.1 |
| Salmonella enterica subspecies I serovar Typhimurium DT104 | NC_022569.1 | Vibrio vulnificus YJ016 cII | NC_005140.1 |
| Salmonella enterica subspecies I serovar Typhimurium DT2 | NC_022544.1 | Yersinia pseudotuberculosis IP31758 | NC_009708.1 |
| Salmonella enterica subspecies I serovar Typhimurium LT2 | NC_003197.2 | Yersinia pseudotuberculosis IP32953 | NC_006155.1 |
| Salmonella enterica subspecies I serovar Typhimurium SL1344 | NC_016810.1 | Yersinia pseudotuberculosis NCTC3571 | LR134306.1 |
| Salmonella enterica subspecies I serovar Typhimurium str798 | NC_017046.1 | | |
| Salmonella enterica subspecies I serovar Typhimurium strST4/74 | NC_016857.1 | | |
| Salmonella enterica subspecies I serovar Typhimurium strT000240 | NC_016860.1 | | |
| Salmonella enterica subspecies I serovar Typhimurium strU288 | NC_021151.1 | | |
| Salmonella enterica subspecies I serovar Typhimurium UK-1 | NC_016863.1 | | |
| Salmonella enterica subspecies I serovar Typhimurium var5- | NC_021814.1 | | |
| Salmonella enterica subspecies I serovar Typhimurium YU15 | CP014358.1 | | |
