# Supplementary material for: AT Homopolymer Strings in Salmonella enterica Subspecies I Contribute to Speciation and Serovar Diversity
Source: Microorganisms. 2021 Oct 1;9(10):2075. doi: 10.3390/microorganisms9102075 (PMC8538453; doi:10.3390/microorganisms9102075)
Supplement: Supplementary file 1 [file microorganisms-09-02075-s001.zip › Table S2 location of ATmers in Typhimurium LT2.pdf]

TABLE S2. Location and classification of AT 8+mers in *S. enterica* Typhimurium LT2

Category of AT 8+mer	Start of AT 8+mer	End of AT 8+mer	Length	Direction	Distance between start of AT 8+mers
intergenic	303	310	8	reverse	
STM0004	3819	3826	8	reverse	3516
intergenic	11498	11505	8	forward	7679
STM0022	25115	25122	8	forward	13617
STM0071	82574	82581	8	reverse	57459
STM0074	88156	88163	8	forward	5582
STM0084	95968	95975	8	forward	7812
intergenic	100814	100821	8	reverse	4846
intergenic	138203	138210	8	reverse	37389
intergenic	173265	173272	8	reverse	35062
intergenic	209196	209203	8	reverse	35931
intergenic	229717	229724	8	forward	20521
intergenic	230227	230234	8	forward	510
intergenic	230254	230261	8	reverse	27
intergenic	237151	237159	9	forward	6897
STM0212	248073	248080	8	reverse	10921
STM0229	268114	268122	9	forward	20041
intergenic	274048	274055	8	reverse	5933
STM0248	288370	288377	8	forward	14322
intergenic	304384	304391	8	forward	16014
intergenic	304417	304424	8	reverse	33
STM0290	332273	332280	8	forward	27856
STM0319	364624	364632	9	reverse	32351
STM0333	376854	376861	8	reverse	12229
STM0341	385921	385928	8	forward	9067
STM0359	408637	408644	8	forward	22716
intergenic	417817	417825	9	reverse	9180
STM0397-regulon	450719	450727	9	reverse	32901
STM0437	490762	490769	8	reverse	40042
intergenic	503022	503029	8	reverse	12260
intergenic	515088	515095	8	reverse	12066
STM0497	557229	557236	8	reverse	42141
intergenic	580065	580072	8	reverse	22836
STM0528	590722	590729	8	reverse	10657
STM0551	612356	612363	8	forward	21634
STM0555	614167	614174	8	reverse	1811
STM0558	616481	616488	8	reverse	2314
intergenic	621689	621696	8	reverse	5208
STM0626-regulon	689650	689657	8	forward	67961
intergenic	693173	693180	8	reverse	3523
intergenic	694829	694836	8	reverse	1656
intergenic	698315	698322	8	forward	3486
STM0649	712001	712008	8	forward	13686

STM0664-regulon	728765	728772	8	forward	16764
intergenic	747296	747303	8	reverse	18531
STM0720	785218	785225	8	forward	37922
STM0722	787718	787725	8	reverse	1439
STM0723	788256	788263	8	forward	538
STM0756	820877	820884	8	forward	32621
intergenic	823058	823065	8	reverse	2181
STM0795	861570	861577	8	forward	38512
intergenic	863944	863952	9	reverse	2374
intergenic	866685	866692	8	forward	2740
intergenic	874135	874142	8	reverse	7450
STM0810	875698	875705	8	forward	1563
STM0827	894495	894502	8	reverse	18797
intergenic	899265	899272	8	forward	4770
intergenic	900093	900100	8	reverse	828
STM0858	931603	931610	8	forward	31510
STM0870	944140	944147	8	forward	12537
intergenic	976389	976396	8	forward	32249
intergenic	992874	992882	9	reverse	16485
STM0929	1004180	1004187	8	reverse	11305
intergenic	1005279	1005286	8	forward	1099
intergenic	1027405	1027412	8	forward	22126
intergenic	1091439	1091446	8	forward	64034
STM1006	1099593	1099600	8	reverse	8154
STM1023	1112426	1112433	8	reverse	12833
STM1054	1142433	1142440	8	forward	30007
STM1062	1153616	1153623	8	forward	11183
STM1063	1154307	1154314	8	forward	691
intergenic	1162292	1162299	8	forward	7985
intergenic	1162330	1162337	8	forward	38
STM1106	1193918	1193925	8	forward	31588
STM1107-regulon	1194017	1194024	8	forward	99
STM1130	1219764	1219771	8	forward	25747
STM1135	1224894	1224901	8	forward	5130
intergenic	1230786	1230793	8	forward	5892
intergenic	1232116	1232123	8	reverse	1330
STM1157	1245414	1245421	8	reverse	13298
STM1163-regulon	1249624	1249631	8	forward	4210
STM1169	1254099	1254106	8	forward	3900
intergenic	1296516	1296523	8	reverse	42417
STM1224	1310635	1310642	8	forward	14119
STM1240	1327591	1327598	8	reverse	16956
STM1240	1327665	1327672	8	reverse	74
STM1240	1327695	1327702	8	forward	30
intergenic	1363853	1363860	8	forward	36158
intergenic	1368633	1368640	8	forward	4780
STM1363_regulon	**1444823**	**1444831**	**9**	**forward**	**76190**
intergenic	1450354	1450361	8	forward	5530
intergenic	1459323	1459330	8	forward	8969
intergenic	1475189	1475196	8	reverse	15866
STM1392	1477236	1477243	8	reverse	2047
STM1402	1487337	1487344	8	forward	10101
STM1409	1490407	1490414	8	forward	3070

STM1432	1510741	1510748	8	reverse	20334
STM1432	1510752	1510759	8	reverse	11
intergenic	1526387	1526395	9	forward	15635
intergenic	1535951	1535958	8	reverse	9563
STM1477	1553907	1553914	8	reverse	17956
STM1484	1561666	1561673	8	forward	7759
intergenic	1587885	1587892	8	reverse	26219
intergenic	1593975	1593984	10	reverse	6090
intergenic	1604045	1604052	8	reverse	10068
STM1550	1626757	1626764	8	reverse	22712
STM1554	1630624	1630631	8	reverse	3867
STM1560	1641163	1641170	8	forward	10539
STM1602	1692279	1692286	8	reverse	51116
STM1630	1720502	1720509	8	forward	28223
intergenic	1749925	1749932	8	forward	29423
STM1666	1759274	1759281	8	forward	9349
STM1670	1763943	1763950	8	reverse	4669
STM1698	1791330	1791337	8	reverse	27387
STM1716	1813896	1813903	8	reverse	22566
intergenic	1814270	1814277	8	forward	374
intergenic	1818514	1818521	8	reverse	4244
intergenic	1847730	1847737	8	reverse	29216
intergenic	1862495	1862502	8	forward	14765
STM1798	1897289	1897296	8	forward	34794
intergenic	1909061	1909068	8	reverse	11772
STM1813	1911893	1911900	8	reverse	2832
intergenic	1915168	1915175	8	forward	3275
intergenic	1937638	1937645	8	reverse	22470
intergenic	1941093	1941101	9	forward	3455
STM1854	1951706	1951713	8	reverse	10612
intergenic	1952885	1952892	8	reverse	1179
STM1862	1959668	1959675	8	forward	6783
STM1869	1965921	1965928	8	reverse	6253
intergenic	1981357	1981364	8	forward	15436
STM1887	1981864	1981871	8	forward	507
STM1889	1984944	1984951	8	reverse	3080
intergenic	2021075	2021082	8	forward	36131
STM1939	2032731	2032738	8	reverse	11656
STM1940	2034236	2034243	8	reverse	1505
STM1941	2034981	2034988	8	forward	745
STM1963	2051934	2051941	8	forward	16953
intergenic	2077259	2077266	8	reverse	25325
STM2020	2101583	2101590	8	reverse	24324
STM2065	2139855	2139862	8	reverse	38272
intergenic	2149557	2149565	9	reverse	9702
intergenic	2159312	2159319	8	forward	9754
STM2082	2162284	2162291	8	forward	2972
STM2086	2166754	2166761	8	reverse	4470
STM2088	2169612	2169619	8	forward	2858
STM2090	2172027	2172034	8	reverse	2415
STM2093	2174824	2174831	8	reverse	2797
STM2097	2178207	2178214	8	reverse	3383
STM2100	2181955	2181962	8	reverse	3748

STM2112	2195935	2195942	8	forward	13980
STM2113	2197346	2197353	8	reverse	1411
STM2118-regulon	2202762	2202769	8	forward	5416
STM2129	2222458	2222465	8	forward	19696
STM2134	2226782	2226789	8	forward	4324
STM2137	2232015	2232022	8	forward	5233
intergenic	2298569	2298576	8	forward	66554
intergenic	2328601	2328608	8	forward	30032
STM2241	2341488	2341495	8	forward	12887
intergenic	2343861	2343868	8	reverse	2373
STM2245	2345894	2345901	8	forward	2033
STM2268-regulon	2366997	2367004	8	reverse	21103
STM2273	2377913	2377920	8	reverse	10916
STM2274	2378993	2379000	8	forward	1080
STM2238	2439576	2439583	8	forward	58441
STM2386	2497010	2497017	8	forward	57434
STM2397	2509762	2509769	8	reverse	12752
STM2403	2518663	2518670	8	reverse	8901
STM2449	2559838	2559845	8	reverse	41175
STM2475	2585641	2585648	8	reverse	25803
STM2490	2605014	2605021	8	reverse	17933
intergenic	2606589	2606596	8	forward	1575
STM2494	2607985	2607992	8	forward	1396
intergenic	2611987	2611994	8	forward	4002
STM2498	2612693	2612700	8	forward	706
intergenic	2612956	2612963	8	forward	263
STM2584	**2730097**	**2730104**	**8**	**reverse**	**117141**
STM2592	2742731	2742738	8	forward	12634
STM2609	2758016	2758024	9	reverse	15285
intergenic	2802005	2802012	8	forward	43988
intergenic	2826350	2826357	8	forward	24345
STM2691	2841501	2841508	8	forward	15151
STM2706	2854470	2854477	8	reverse	12969
intergenic	2885676	2885683	8	forward	31206
intergenic	2888236	2888244	9	forward	2560
intergenic	2888289	2888296	8	forward	52
STM2754	2892589	2892596	8	forward	4300
STM2766	2908226	2908233	8	reverse	15637
intergenic	2910508	2910515	8	forward	2282
STM2782	2930398	2930405	8	forward	19890
STM2819	2968642	2968649	8	forward	38244
STM2862	3007511	3007518	8	forward	38869
STM2878	3022893	3022900	8	reverse	15382
STM2886	3031073	3031080	8	forward	8180
STM2897	3041386	3041393	8	reverse	10313
STM2898	3043091	3043098	8	reverse	1705
intergenic	3044283	3044290	8	reverse	1192
STM2902	3045870	3045877	8	forward	1587
intergenic	3065455	3065462	8	forward	19585
STM2932	3072146	3072153	8	forward	6691
intergenic	3086949	3086956	8	forward	14803
STM2945	3088003	3088010	8	forward	1054
intergenic	3095426	3095433	8	reverse	7423

intergenic	3101710	3101718	9	forward	6284
STM2966	3116704	3116711	8	forward	14993
intergenic	3135521	3135528	8	reverse	18817
intergenic	3157974	3157981	8	forward	22453
STM3005	3164026	3164033	8	forward	6052
intergenic	3170128	3170135	8	forward	6102
intergenic	3170329	3170337	9	forward	201
STM3022	3183503	3183510	8	forward	13173
STM3023	3183980	3183987	8	reverse	477
STM3034	3195767	3195774	8	forward	11787
intergenic	3224585	3224592	8	forward	28818
STM3118	3276846	3276853	8	reverse	52261
STM3121	3280240	3280247	8	reverse	3394
intergenic	3317570	3317577	8	reverse	37330
STM3185	3348286	3348293	8	forward	30716
intergenic	3362464	3362471	8	forward	14178
intergenic	3392257	3392268	12	reverse	29793
STM3233	3398919	3398926	8	forward	6658
intergenic	3404719	3404726	8	reverse	5800
STM3274	3440410	3440417	8	forward	35691
intergenic	3461720	3461727	8	forward	21310
intergenic	3473219	3473226	8	forward	11499
STM3311	3478142	3478149	8	reverse	4923
STM3328	3490502	3490509	8	reverse	12360
intergenic	3501757	3501764	8	forward	11255
intergenic	3560947	3560955	9	reverse	59190
intergenic	3597913	3597920	8	forward	36965
STM3452	3604603	3604610	8	forward	6690
intergenic	3604838	3604845	8	forward	235
intergenic	3626427	3626434	8	reverse	21589
STM3480	3636018	3636025	8	forward	9591
STM3484	3639524	3639531	8	reverse	3506
STM3515	3676865	3676872	8	forward	37341
intergenic	3715413	3715421	9	forward	38548
intergenic	3715555	3715562	8	forward	141
intergenic	3716294	3716302	9	reverse	739
intergenic	3727793	3727800	8	forward	11498
intergenic	3733712	3733719	8	forward	5919
intergenic	3774475	3774482	8	forward	40763
STM3638	3826424	3826432	9	reverse	51949
intergenic	3828296	3828303	8	forward	1871
intergenic	3836708	3836715	8	reverse	8412
STM3658	3843569	3843576	8	reverse	6861
STM3674	3861984	3861991	8	reverse	18415
intergenic	3880285	3880292	8	forward	18301
STM3691	3881321	3881328	8	forward	1036
STM3697	3891460	3891467	8	forward	10139
intergenic	3897443	3897450	8	forward	5983
intergenic	3910767	3910774	8	forward	13324
intergenic	3926659	3926666	8	forward	15892
intergenic	3991176	3991183	8	forward	64517
STM3794	3995143	3995150	8	reverse	3967
STM3806	4007348	4007355	8	forward	12205

intergenic	4010490	4010497	8	reverse	3142
STM3825	4026356	4026363	8	forward	15866
intergenic	4073634	4073642	9	forward	47278
intergenic	4081158	4081165	8	reverse	7523
intergenic	4081169	4081176	8	reverse	11
intergenic	4095558	4095565	8	forward	14389
intergenic	4105484	4105492	9	reverse	9926
intergenic	4108895	4108902	8	reverse	3410
STM3914	4124625	4124632	8	reverse	15730
intergenic	**4209022**	**4209029**	**8**	**forward**	**84397**
STM4039	4250074	4250081	8	reverse	41052
intergenic	4250202	4250209	8	forward	128
intergenic	4263304	4263311	8	reverse	13102
intergenic	4271167	4271174	8	reverse	7863
STM4076	4286594	4286601	8	reverse	15427
STM4086	4295849	4295856	8	reverse	9255
intergenic	4306000	4306007	8	forward	10151
intergenic	4342879	4342887	9	forward	36879
STM4196	**4418289**	**4418297**	**9**	**reverse**	**75409**
STM4260	4481627	4481634	8	forward	63337
intergenic	4502109	4502116	8	reverse	20482
STM4266-regulon	4504400	4504407	8	forward	2291
intergenic	4506004	4506011	8	reverse	1604
intergenic	4538693	4538700	8	forward	32689
STM4314	4560794	4560801	8	reverse	22101
intergenic	4561971	4561978	8	forward	1177
intergenic	4574663	4574670	8	reverse	12692
STM4351	4596149	4596157	9	reverse	21486
intergenic	4614384	4614392	9	reverse	18234
STM4401	4638395	4638402	8	reverse	24010
intergenic	4643638	4643646	9	forward	5243
STM4435	4679637	4679644	8	forward	35998
intergenic	4704946	4704954	9	forward	25309
STM4485	4729445	4729452	8	forward	24498
STM4495	4744842	4744849	8	reverse	15397
STM4524	4778396	4778403	8	reverse	1611
STM4585-regulon	4842031	4842038	8	forward	63635
intergenic	4843637	4843644	8	reverse	1606
STM4600	4857407	4857414	8	forward	13770

average distance between starts	16,450.2
STDev	17,325.3
range	11-117,141
median	11,498.0
number	294
intergenic	131
genes	153
regulons	10
