# Supplementary material for: AT Homopolymer Strings in Salmonella enterica Subspecies I Contribute to Speciation and Serovar Diversity
Source: Microorganisms. 2021 Oct 1;9(10):2075. doi: 10.3390/microorganisms9102075 (PMC8538453; doi:10.3390/microorganisms9102075)
Supplement: Supplementary file 1 [file microorganisms-09-02075-s001.zip › Table S3.pdf]

TABLE S3. Categories of genes from 3 serovars of *S. enterica* subspecies I that vary in AT 8+mer mutations[1]

STM gene accession	SEEG gene accession	SEN gene accession	AT 8+mer Sequence variation	Common name of gene	Description of target gene	Gene function
A. Genes of *S.* Typhimurium (STM) that vary from *S.* Gallinarum (SEEG) and/or *S.* Enteritidis (SEN)						
STM0105	SEEG9184_RS20395	SEN_RS00535	STM 1 bp sub	yabI	All ORFs intact	DedA family protein
STM0212	---	SEN_RS01100	STM 1 bp sub	unnamed	Both ORFs intact	MFS transporter
STM0229	SEEG9184_RS19720	SEN_RS01185	SEEG, SEN 1 bp sub	lpxB	All ORFs intact	Lipid-A-disaccharide synthase
STM0341	SEEG9184_RS23875	SEN_RS01660	SEEG 1 bp del	unnamed	SEEG pseudogene	putative inner membrane protein
STM0342	SEEG9184_RS2540	SEN_RS24170	SEN 1 bp insert	unnamed	All ORFs intact	putative periplasmic protein
STM0555	SEEG9184_RS23515	SEN_RS24190	SEEG, SEN 1 bp sub	unnamed	All pseudogenes	pseudogene in all 3 genomes; frameshift relative to E coli ES3 putative transposase
STM0558	---	SEN_RS02735	SEN 1 bp del	yfdH	Both ORFs intact	putative glycosyltransferase
STM1107_regulon	SEEG9184_RS15835_regulon	SEN_RS05020_regulon	STM 1 SNP del	hpaX-regulon	na	4-hydroxyphenylacetate permease
STM1107	SEEG9184_RS15835	SEN_RS05020	same	hpaX	All ORFs intact	4-hydroxyphenylacetate permease
STM1130	SEEG9184_RS15695	---	SEEG 1 bp del	unnamed	SEEG pseudogene	N-acetylneuraminic acid mutarotase
STM1520	SEEG9184_RS12690	---	STM 1 bp sub	marR	Both ORFs intact	transcriptional regulator multiple antibiotic resistance protein MAR
STM1550	---	SEN_RS07790	Deletion of 154 bp in SEN	unnamed	SEN pseudogene	type II toxin-antitoxin system mRNA interferase toxin
STM1602	SEEG9184_RS13085	---	SEEG 1 bp del	sifB	SEEG pseudogene	effector protein SifB
STM1635	SEEG9184_RS13270	---	STM 1 bp sub	unnamed	ORF intact	amino acid ABC transporter ATP-binding protein
STM1670	SEEG9184_RS13465	---	SEEG 1 bp del	unnamed	ORF intact	HO protein
STM1698	SEEG9184_RS13605	---	SEEG 1 bp del	steC	SEEG pseudogene	secreted effector kinase SteC
STM1869	SEEG9184_RS14565	---	SEEG 1 bp del	unnamed	SEEG pseudogene	HO protein
STM1939	SEEG9184_RS15200	---	SEEG 1 bp del	unnamed	SEEG pseudogene	putative glucose-6-phosphate dehydrogenase
STM1941	SEEG9184_RS15210	SEN_RS05525	SEEG, SEN 1 bp del	unnamed	SEEG pseudogene	HO protein
STM2047	SEEG9184_RS10150	SEN_RS10650	STM 1 bp sub	pduL	All ORFs intact	propanediol utilization protein
STM2090	SEEG9184_RS10005	SEN_RS10870	SEEG, SEN 1 bp sub	rfbH	All ORFs intact	lipopolysaccharide biosynthesis protein; LPS side chain defect
STM2100	SEEG9184_RS09955	SEN_RS10920	SEEG, SEN 1 bp sub	wcaL	All ORFs intact	colanic acid biosynthesis glycosyltransferase
STM2129	SEEG9184_RS09800	---	SEEG 1 bp del	yegB	SEEG pseudogene	multidrug transporter subunit MdtD
STM2134	SEEG9184_RS09775	---	SEEG 1 bp del	unnamed	ORF intact	HO protein
STM2268_regulon	bp1841830-1842036	bp2360965-2361170	SEN 1 SNP del	micF-regulon	na	Not annotated in SEEG:ribosomal binding site of micF in STM
STM2277_regulon	SEEG9184_RS09060	---	SEEG 1 SNP del	ubiG-regulon	na	3-demethylubiquinone-9 3-methyltransferase
STM3022	---	SEN_RS14915	SEN 1 bp sub	unnamed	ORF intact	transporter
STM3166	SEEG9184_RS04980	SEN_RS15645	STM 1 bp sub	unnamed	All ORFs intact	putative cation transporter
STM3212	SEEG9184_RS04730	---	STM 1 bp sub	mug	ORF intact	double-stranded uracil-DNA glycosylase
STM3464	SEEG9184_RS01925	---	STM 1 bp del	prkB	ORF intact	putative phosphoribulokinase
STM3638	SEEG9184_RS01040	---	SEEG 1 bp del	lpfC	ORF intact	fimbrial assembly protein; SEEG9184 has 5 bp deletion
STM3674	SEEG9184_RS00845	SEN_RS18190	SEEG, SEN 1 bp sub	lyxK	SEEG pseudogene	carbohydrate kinase
STM4351	---	SEN_RS21445	SEN 1 bp del	unnamed	ORF intact	arginine ABC transporter substrate-binding protein
B. Genes of *S.* Typhimurium (STM) with AT 8+mers but with no homolog to either *S*. Gallinarum or *S.* Enteritidis						
STM0290	---	---	na	unnamed	ORF intact	HO protein
STM0720	---	---	na	unnamed	ORF intact	putative glycosyl transferase
STM0722	---	---	na	unnamed	ORF intact	ABC transporter permease
STM0723	---	---	na	unnamed	ORF intact	similar to E coli ATP-binding component of putrescine transport system; sugar ABC transporter ATP-binding protein
STM0870	---	---	na	unnamed	ORF intact	putative inner membrane protein
STM0929	---	---	na	unnamed	ORF intact	Gifsy-2 prophage excisionase
STM1006	---	---	na	unnamed	ORF intact	Gifsy-2 prophage protein
STM1023	---	---	na	unnamed	ORF intact	Gifsy-2 prophage protein
STM1554	---	---	na	unnamed	ORF intact	putative coiled-coil protein
STM2088	---	---	na	rfbX	ORF intact	O-antigen transferase:LPS side chain defect if mutated; putative transporter
STM2137	---	---	na	unnamed	ORF intact	putative cytoplasmic protein
STM2584	---	---	na	gogB	ORF intact	type III secretion system protein
STM2592	---	---	na	unnamed	ORF intact	similar to phage tail component L
STM2609	---	---	na	unnamed	ORF intact	Gifsy-1 prophage protein
STM2706	---	---	na	unnamed	ORF intact	Fels-2 prophage protein; similar to tail fiber protein in phage P2
STM2754	---	---	na	unnamed	ORF intact	hexulose-6-phosphate synthase
STM2766	---	---	na	unnamed	ORF intact	HO protein
STM2902	---	---	na	unnamed	ORF intact	putative cytoplasmic protein
STM4435	---	---	na	unnamed	ORF intact	inosose isomerase
STM4495	---	---	na	unnamed	ORF intact	type II restriction enzyme methylase subunit
STM4524	---	---	na	hsdS	ORF intact	restriction endonuclease subunit S
C. Genes of S. Enteritidis and/or *S.* Gallinarum with similar AT 8+mers but with no homolog in *S.* Typhimurium						
---	SEEG9184_RS10025	SEN_RS10850	same	unnamed	Both ORFs intact	glycosyltransferase family 2 protein
---	SEEG9184_RS10420	SEN_RS10400	same	unnamed	Both ORFs intact	HO protein
---	SEEG9184_RS10430	SEN_RS10390	same	unnamed	Both ORFs intact	SLATT domain-containing protein
---	SEEG9184_RS10595	SEN_RS09970	same	unnamed	Both ORFs intact	HO protein
---	SEEG9184_RS14095	SEN_RS06485	same	unnamed	Both ORFs intact	HO protein
---	SEEG9184_RS14610	---	na	unnamed	ORF intact	HO protein
---	SEEG9184_RS15540	---	na	unnamed	ORF intact	HO protein
---	SEEG9184_RS19390	---	na	unnamed	Both ORFs intact	DUF3987 domain-containing protein
---	SEEG9184_RS19405	---	na	unnamed	Both ORFs intact	HO protein
---	SEEG9184_RS21290	SEN_RS22310	same	unnamed	Both ORFs intact	AAA family ATPase
---	SEEG9184_RS21300	SEN_RS22300	same	unnamed	Both ORFs intact	restriction endonuclease subunit M
---	SEEG9184_RS21305_1,2,3,4	SEN_RS22295_1,2,3,4	same	unnamed	Both ORFs intact	N-6 DNA methylase
---	SEEG9184_RS21500_1,2_regulon	SEN_RS22090_1,2_regulon	(1) SEN 1 SNP sub, (2) same	unnamed	na	AraC family transcriptional regulator
---	SEEG9184_RS21505	SEN_RS22085	SEEG 1 SNP del	sefD	SEEG pseudogene	adhesin
---	SEEG9184_RS21510_1,2,3 (PS)	SEN_RS22080_1,2,3	same	sefC	SEEG pseudogene	outer membrane fimbrial user protein SefC, pseudo in SG, which has an extra A/T to make a 7mer
---	SEEG9184_RS22845	SEN_RS20760	same	unnamed	ORF intact	putative inner membrane protein
D. Genes of *S.* Typhimurium and *S.* Enteritidis with AT 8+mers but no homolog in *S.* Gallinarum						

STM0870	---	SEN_RS04220	same	unnamed	Both ORFs intact	protein transporter: not in SEEG9184; see SG_RS04310
STM1054	---	SEN_RS23040	same	unnamed	SEN pseudogene	Gifsy-2 prophage protein in STM: GC rich region has a deletion in in SEN due to 7bp insert in a guanidine rich fragment, causing a frameshift
STM1666	---	SEN_RS07090	same	unnamed	STM pseudogene	STM has in-frame stop following codon 24; SEN, hypothetical protein
STM2065	---	SEN_RS10740	same	phsA	Both ORFs intact	thiosulfate reductase
STM3023	---	SEN_RS14920	same	yohL/rcnR	Both ORFs intact	transcriptional regulator; Ni(ii)/Co(ii)-binding transcriptional repressor
STM3034	---	SEN_RS14975	same	vapB	Both ORFs intact	toxin-antitoxin system antitoxin VapB

E. Genes of *S.* Typhimurium (STM), *S.* Gallinarum (SEEG), and *S.* Enteritidis with conserved AT 8+mers

STM0004	SEEG9184_RS20920	SEN_RS00020	same	thrC	All ORFs intact	threonine synthase
STM0022	SEEG9184_RS20830	SEN_RS00110	same	bcfB	All ORFs intact	fimbrial chaperone protein
STM0071	SEEG9184_RS20580	SEN_RS00360	same	caiC	SEEG pseudogene	crotonobetaine/carnitine-CoA ligase
STM0074	SEEG9184_RS20565	SEN_RS00375	same	caiT	All ORFs intact	L-carnitine:gamma-butyrobetaine antiporter
STM0084	SEEG9184_RS20510	SEN_RS00430	same	unnamed	All ORFs intact	phosphatase; sulfatase-like hydrolase/transferase
STM0248	SEEG9184_RS19625	SEN_RS01280	same	yaeD/gmhB	All ORFs intact	D-glycero-beta-D-manno-heptose 1,7-bisphosphate-7-phosphatase
STM0319	SEEG9184_RS19215	SEN_RS01550	same	crl	All ORFs intact	sigma factor-binding protein; curli surface fiber csgA regulation
STM0333	SEEG9184_RS19130	SEN_RS01620	same	unnamed	All ORFs intact	putative LysR family transcriptional regulator
STM0359	SEEG9184_RS23530	SEN_RS01755	same	unnamed	All ORFs intact	HO protein
STM0397_regulon	SEEG9184_RS18805_regulon	SEN_RS01950_regulon	same	phoB-regulon	na	phosphate response regulator transcription factor
STM0437	SEEG9184_RS18595	SEN_RS02150	same	unnamed	All ORFs intact	sel1 repeat family protein
STM0497	SEEG9184_RS18275	SEN_RS02445	same	unnamed	All ORFs intact	HO protein
STM0528	SEEG9184_RS18125	SEN_RS02595	same	allD	All ORFs intact	ureidoglycolate dehydrogenase
STM0551	SEEG9184_RS18005	SEN_RS02710	same	unnamed	All ORFs intact	diguanylate cyclase
STM0626	SEEG9184_RS17645	SEN_RS03035	same	dpiB	All ORFs intact	sensor histidine kinase
STM0664_regulon	SEEG9184_RS17450_regulon	SEN_RS03235_regulon	same	gltJ-regulon	na	glutamate/aspartate ABC transporter permease
STM0756	SEEG9184_RS17055	SEN_RS03640	same	nadA	All ORFs intact	quinolinate synthase
STM0795	SEEG9184_RS16855	SEN_RS03835	same	bioF	All ORFs intact	8-amino-7-oxononanoate synthase
STM0810	SEEG9184_RS16775	SEN_RS03910	same	unnamed	All ORFs intact	HO protein
STM0827	SEEG9184_RS16695	SEN_RS03995	same	ybiO	All ORFs intact	mechanosensitive channel protein
STM0858	SEEG9184_RS16515	SEN_RS04155	same	unnamed	SEEG pseudogene	electron transfer flavoprotein-ubiquinone oxidoreductase
STM1062	SEEG9184_RS16060	SEN_RS04790	same	uup	All ORFs intact	ABC transporter ATP-binding protein
STM1063	SEEG9184_RS16055	SEN_RS04795	same	pqiA	All ORFs intact	paraquat-inducible protein A
STM1106	SEEG9184_RS15840	SEN_RS05015	same	hpaI	All ORFs intact	2,4-dihydroxyhept-2-ene-1,7-dioic acid aldolase
STM1157	SEEG9184_RS10755	SEN_RS09830	same	yceI	All ORFs intact	HO protein
STM1163_regulon	SEEG9184_RS10790_regulon	SEN_RS09800_regulon	same	pyrC-regulon	na	dihydrorotase
STM1169	SEEG9184_RS10820	SEN_RS09770	same	mviM	All ORFs intact	virulence factor MviM; Gfo/Idh/MocA family oxidoreductase
STM1224	SEEG9184_RS11110	SEN_RS09490	same	sifA	All ORFs intact	replication in macrophages; SPI-2 type III secretion system effector SifA
STM1363_regulon	SEEG9184_RS11875	SEN_RS22750	same	rprA-regulon	na	regulatory RNA: anti-sense sRNA RprA
STM1392	SEEG9184_RS12025	SEN_RS08580	same	ssrA	All ORFs intact	hybrid sensor histidine kinase/response;two component system sensor kinase
STM1402	SEEG9184_RS12080	SEN_RS08530	same	sseE	All ORFs intact	LcrR family type III secretion system chaperone
STM1409	SEEG9184_RS12115	SEN_RS08495	same	ssaJ	All ORFs intact	EscJ/YscJ/HrcJ family type III secretion inner membrane ring protein
STM1432_1,2	SEEG9184_RS12225_1,2	SEN_RS08380_1,2	same	ydhO	All ORFs intact	putative cell wall-associated hydrolase;C40 family peptidase
STM1477	SEEG9184_RS12450	SEN_RS08155	same	ydgI	All ORFs intact	putative amino acid transporter/permease
STM1484	SEEG9184_RS12495	SEN_RS08120	same	unnamed	All ORFs intact	serine protease
STM1560	SEEG9184_RS12850	SEN_RS07750	same	treZ/treY	SEEG treY pseudogene	malto-oligosyltrehalose trehalohydrolase
STM1630	SEEG9184_RS13245	SEN_RS07380	same	unnamed	All ORFs intact	HO protein
STM1716	SEEG9184_RS13730	SEN_RS06820	same	sohB	All ORFs intact	protease
STM1798	SEEG9184_RS14165	SEN_RS06415	same	ycgR	All ORFs intact	flagellar brake protein
STM1813	SEEG9184_RS14230	SEN_RS06340	same	ycgL	All ORFs intact	putative cytoplasmic protein
STM1854	SEEG9184_RS23435	SEN_RS06135	same	unnamed	All ORFs intact	HO protein; membrane protein
STM1862	SEEG9184_RS14495	SEN_RS06085	same	pagO	All ORFs intact	EamA/DMT family transporter (PAGO protein)
STM1887	SEEG9184_RS14940	SEN_RS05795	same	yebK	All ORFs intact	transcriptional regulator of HexR;MurR/RpiR family transcriptional regulator
STM1889	SEEG9184_RS14950	SEN_RS05785	same	lpxM	All ORFs intact	lauroyl-Kdo(2)-lipid IV(A) myristoyltransferase
STM1940	SEEG9184_RS15205	SEN_RS05530	same	unnamed	All ORFs intact	putative cell wall-associated hydrolase
STM1963	SEEG9184_RS15315	SEN_RS05415	same	amyA	All ORFs intact	cytoplasmic alpha-amylase
STM2020	SEEG9184_RS23790	SEN_RS10515	same	cbiO	SEEG pseudogene	cobalt transport atp-binding protein CbiO: B12 synthesis associated?
STM2082	SEEG9184_RS10050	SEN_RS10825	same	rfbP	All ORFs intact	UDP-phosphate galactose phosphotransferase; bifunctional enzyme; LPS side chain defect
STM2086	SEEG9184_RS10030	SEN_RS10845	same	rfbU	All ORFs intact	mannosyl transferase
STM2093	SEEG9184_RS09990	SEN_RS10885	same	rfbI	All ORFs intact	CDP-6-deoxy-delta-3,4-glucoseen reductase; LPS
STM2097	SEEG9184_RS09970	SEN_RS10905	same	rfbB	All ORFs intact	dTDP-glucose 4,6-dehydratase
STM2112	SEEG9184_RS09895	SEN_RS10980	same	wcaD	All ORFs intact	colanic acid polymerase WcaD
STM2113	SEEG9184_RS09890	SEN_RS10985	same	wcaC	All ORFs intact	colanic acid biosynthesis glycosyltransferase
STM2118	SEEG9184_RS09865	SEN_RS11010	same	wza	All ORFs intact	polysaccharide exporter
STM2118_regulon	not annotated_regulon	SEN_RS11010_regulon	same	STM2118-regulon	na	see STM2118
STM2241	SEEG9184_RS09260	SEN_RS11570	same	sspH2	SEEG pseudogene	E3 ubiquitin--protein ligase; induced by the SPI-2 regulatory ssrA/B
STM2245	SEEG9184_RS09225	SEN_RS11595	same	unnamed	All ORFs intact	putative outer membrane protein
STM2273	SEEG9184_RS09075	SEN_RS11730	same	unnamed	All ORFs intact	MR-MLE family protein: starvation sensing protein?
STM2274	SEEG9184_RS09070	SEN_RS11735	same	unnamed	SEEG pseudogene	MFS transporter
STM2328	SEEG9184_RS08795	SEN_RS12005	same	nuoA	All ORFs intact	NADH-quinone oxidoreductase subunit A
STM2386	SEEG9184_RS08495	SEN_RS12305	same	smrB	All ORFs intact	endonuclease
STM2397	SEEG9184_RS08410	SEN_RS12380	same	pgtB	All ORFs intact	sensor histidine kinase
STM2403	SEEG9184_RS08370	SEN_RS12420	same	glk	All ORFs intact	glucokinase
STM2449	SEEG9184_RS08135	SEN_RS12660	same	unnamed	All ORFs intact	putative acetyltransferase
STM2475	SEEG9184_RS08005	SEN_RS12780	same	unnamed	All ORFs intact	HO protein
STM2490	SEEG9184_RS07925	SEN_RS12855	same	gcvR	All ORFs intact	transcriptional repressor of gcv operon
STM2494	SEEG9184_RS07895	SEN_RS12885	same	unnamed	All ORFs intact	HO protein
STM2498_regulon	SEEG9184_RS07875	SEN_RS12905	same	upp-regulon	na	uracil phosphoribosyltransferase
STM2691	SEEG9184_RS07115	SEN_RS13585	same	unnamed	SEEG pseudogene	type I secretion system permease/ATPase: TolC family OMP
STM2782	SEEG9184_RS07005	SEN_RS13665	same	mig-14	All ORFs intact	antimicrobial resistance protein Mig-14
STM2819	SEEG9184_RS06805	SEN_RS13870	same	yqaA/yqaB	SEEG yqaB pseudogene	HO protein
STM2862	SEEG9184_RS06575	SEN_RS14085	same	sitB	All ORFs intact	manganese/iron transporter ATP-binding protein
STM2878	SEEG9184_RS06495	SEN_RS14165	same	sptP	All ORFs intact	pathogenicity island 1 effector protein StpP

STM2886	SEEG9184_RS06455	SEN_RS14205	same	sicA	All ORFs intact	CesD/SycD/LcrH family type III secretion system chaperone: surface presentation of antigens
STM2897	SEEG9184_RS06400	SEN_RS14260	same	invE	All ORFs intact	type III secretion system gatekeeper InvE
STM2898	SEEG9184_RS06395	SEN_RS14265	same	invG	All ORFs intact	type III secretion system outer membrane ring protein InvG
STM2932	SEEG9184_RS06225	SEN_RS14430	same	ygbE	All ORFs intact	putative inner membrane protein
STM2945	SEEG9184_RS06150	SEN_RS14495	same	sopD	All ORFs intact	SPI-1 type III secretion system effector
STM2966_regulon	SEEG9184_RS05995	SEN_RS23740	same	csrB-regulon	na	regulatory RNA in LT2
STM3005	SEEG9184_RS05790	SEN_RS14830	same	mutH	All ORFs intact	DNA mismatch repair endonuclease MutH
STM3118	SEEG9184_RS05225	SEN_RS15405	same	unnamed	All ORFs intact	putative acetyl-CoA hydrolase
STM3121	SEEG9184_RS05210	SEN_RS15420	same	unnamed	All ORFs intact	putative LysR family transcriptional regulator
STM3185	SEEG9184_RS04880	SEN_RS15745	same	yqiE	All ORFs intact	ADP-ribose diphosphatase
STM3233	SEEG9184_RS04620	SEN_RS15990	same	yqjG	All ORFs intact	glutathione-dependent reductase
STM3274	SEEG9184_RS04430	SEN_RS16165	same	yhbU	All ORFs intact	putative protease
STM3311	SEEG9184_RS04240	SEN_RS16355	same	yrbD/mlaD	All ORFs intact	outer membrane lipid asymmetry maintenance protein MlaD
STM3328	SEEG9184_RS04150	SEN_RS16440	same	arcB	All ORFs intact	two-component sensor histidine kinase; senses redox conditions
STM3480	SEEG9184_RS01850	SEN_RS17205	same	yhfL	All ORFs intact	DUF4223 domain-containing protein
STM3484	SEEG9184_RS01830	SEN_RS17225	same	dam	All ORFs intact	DNA adenine methylase
STM3515_regulon	SEEG9184_RS01665_regulon	SEN_RS17380_regulon	same	malT-regulon	na	transcriptional regulator
STM3658	SEEG9184_RS00930	SEN_RS18105	same	yiaH	SEEG pseudogene	acetyltransferase
STM3691	SEEG9184_RS00750	SEN_RS18275	same	sadA	All ORFs intact	trimeric autotransporter adhesin; SEEG has 42 bp deletion, no AT 8+mer involved
STM3697_regulon	SEEG9184_RS00715_regulon	SEN_RS18305_regulon	same	unnamed	na	RBS for mandelate racemase/muconate lactonizing protein
STM3794	SEEG9184_RS00245	SEN_RS18745	same	tsx, nupG E. coli	All ORFs intact	DeoR family transcriptional regulator
STM3806	bp37560-37846	bp3881833-3882117	same	unnamed	All pseudogenes	not annotated in SEN or SEEG; pseudogene in STM; frameshift
STM3825	SEEG9184_RS00075	SEN_RS18905	same	torT	All ORFs intact	TMAO reductase system periplasmic protein
STM3914	SEEG9184_RS03405	SEN_RS19330	same	rhlB	All ORFs intact	ATP-dependent RNA helicase RhlB
STM4039	SEEG9184_RS23265	SEN_RS24515	same	unnamed	EN, SEEG pseudogene	HO protein
STM4076	SEEG9184_RS02975	SEN_RS20130	same	ydeZ/lsrD	All ORFs intact	putative sugar transport protein
STM4086	SEEG9184_RS03025	SEN_RS20180	same	glpK	All ORFs intact	glycerol kinase
STM4196	SEEG9184_RS22850	SEN_RS20755	same	unnamed	All ORFs intact	HO protein
STM4260	SEEG9184_RS22640	SEN_RS20975	same	unnamed	All ORFs intact	predicted cation efflux pump; HLYD secretioncation transporter
STM4266	SEEG9184_RS22610	SEN_RS21005	same	soxR	All ORFs intact	redox-sensitive transcriptional activator SoxR: contains iron-sulfur center for redox-sensing (MerR family)
STM4314	SEEG9184_RS22360	SEN_RS21255	same	unnamed	All ORFs intact	putative luxR family bacterial regulatory
STM4401	SEEG9184_RS21915	SEN_RS21705	same	ytfG	All ORFs intact	NAD(P)-dependent oxidoreductase
STM4485	SEEG9184_RS21575	SEN_RS22030	same	idnK	All ORFs intact	gluconate kinase
STM4585	SEEG9184_RS21020	SEN_RS22580	same	gpmB	All ORFs intact	phosphoglycerate mutase
STM4585_regulon	SEEG9184_RS21020_regulon	SEN_RS22580_regulon	same	gpmB	na	phosphoglycerate mutase regulon
STM4600	SEEG9184_RS20940	SEN_RS22655	same	lasT	All ORFs intact	tRNA/rRNA methyltransferase

[1]Results are from comparing 3 genomes, namely S. Typhimurium NC_003197.2 (STM), S. Enteritidis NC_011294.1 (SEN), and S. Gallinarum CP019035.1 (SEEG).

[2]Genes of STM that vary in AT 8+mer sequences as compared to SEEG and SEN are listed in both sections A and B.

[3]Abbreviations: na, not applicable; ORF, open reading frame.
